# Antiviral therapy in neonatal cholestatic cytomegalovirus hepatitis

**DOI:** 10.1186/1471-230X-7-9

**Published:** 2007-03-13

**Authors:** Tanju Basarir Ozkan, Resit Mistik, Bunyamin Dikici, Hülya Ozturk Nazlioglu

**Affiliations:** 1 Pediatric Gastroenterology Department, Hepatology and Nutrition, Uludag University Faculty of Medicine, Bursa, Turkey; 2 Clinical Microbiology and Infectious Diseases Department, Uludag University Faculty of Medicine, Bursa, Turkey; 3 Pediatrics Department, Duzce University Faculty of Medicine, Duzce, Turkey; 4 Pathology Department, Uludag University Faculty of Medicine, Bursa, Turkey

## Abstract

**Background:**

Neonatal hepatitis refers to a heterogeneous group of disorders, caused by many factors including cytomegalovirus infection, revealing similar morphologic changes in the liver of an infant less than 3 months of age. Approximately 40% of cholestasis in infants is due to neonatal hepatitis. It may cause latent or acute cholestatic or chronic hepatitis, including cirrhosis in immunocompetant infant.

**Methods:**

Twelve infants diagnosed with neonatal cytomegalovirus hepatitis in the last one year were included in the study. Group 1 consisted of seven babies treated with ganciclovir for 21 days. Group 2 included five cases who did not receive antiviral treatment. Physical examination, biochemical, serologic and virologic tests were done for both groups at the time of diagnosis and in the third month.

**Results:**

Initial levels of total bilirubin, aminotransferases, gamma glutamyl transpeptidase, and alkaline phosphatase revealed a significant decrease after the treatment in Group 1 (p < 0.05) when compared with Group 2. This study revealed that ganciclovir treatment is a safe and effective in cases with cholestatic hepatitis. Similarly, all the patients in the treatment group had evidence of improvement serologically and virologically, while the comparison group did not reveal any significant change(p < 0.01).

**Conclusion:**

The clinical spectrum of perinatal infection varies from an asymptomatic infection or a mild disease to a severe systemic involvement, including central nervous system. The treatment in the early period of infection improved serologic markers and cholestatic parameters significantly. Further studies will lead us to clarify the efficacy of ganciclovir treatment in the early period of cytomegalovirus hepatitis, and the preventive role of anti-viral therapy on progressive liver disease due to cholestasis and hepatitis in neonatal cytomegalovirus infection.

## Background

Neonatal hepatitis is a specific type of hepatitis seen in the first months of life. Hepatitis A-E viruses and other hepatotrophic viruses (Epstein-Barr virus(EBV), herpes viruses, adenoviruses and parvovirus) are known to be the main causes of the disease. It is obvious that A-E hepatotrophic viruses are the main causes of etiology in 10% of acute viral hepatitis cases without immunosuppression. Cytomegalovirus (CMV) is a member of herpes virus family, and although it is known that CMV and other herpes viruses can cause significant pathologies (particularly in immunodeficient patients), they can also affect individuals with normal immune system. Typical acute unicteric hepatitis, as a part of systemic infection, is one of these pathologies [1].

Approximately 20% of children less than 15 years of age and 50–60% of individuals younger than 25–30 years of age are infected with CMV. It is known that virus replicates in both hepatocytes and cholangiocytes during infection. However, controversy exists about the pathogenesis of hepatic disease whether related to the direct cytopathic effect of the virus or the immune response of the host. In addition, the hepatocyte damage in latent infections has not yet been well explained. Besides this, it is obvious that patients with chronic viral hepatitis and cirrhosis may have more sensitivity to acute CMV infections resulting in additional hepatic damage[2-4].

In severe cases, ganciclovir or foscarnet treatment may be effective. Although ganciclovir treatment reported to be effective in CMV retinitis, esophagitis, hepatitis and pneumonia in adults, there is insufficient research in children. It is mentioned that ganciclovir may be used effectively in symptomatic congenital or neonatal CMV infections but its side effects are of concern [3].

In the current study, the objective was to evaluate efficacy of ganciclovir in cholestasis of neonatal CMV hepatitis, which is an important step for prevention of chronic CMV hepatitis.

## Methods

Twelve infants diagnosed with neonatal cytomegalovirus hepatitis in the last one year were included in the study. Local ethics comittee (Abant Izzet Baysal University, Duzce Medical Faculty Ethics Committee) approved the study and the written informed consent obtained from the parents of the patients. The babies (n:7) whose parents accepted ganciclovir therapy formed the treatment group(Group1), while the other five babies without ganciclovir therapy formed the comparison group (Group2) (Table 1). One baby with ileal atresia (because he may have additional enterohepatic circulation disorder) and another one with myoclonic convulsions receiving anticonvulsant therapy (because he may have additional toxic hepatitis) were excluded before the study groups were chosen. Liver biopsy was performed in babies with unexplained hypertransaminasemia, icterus and/or hepatomegaly, in order to reveal idiopathic neonatal hepatitis, extrahepatic bilier atresia, immune insufficiency and metabolic hepatic disorders (such as α_1_antityripsine deficiency, storage disorders) which were also among exclusion criteria. Convulsions, petechia, purpura, chorioretinitis, microcephaly, neuromuscular dysfunction, cerebral calcifications, and/or severe disease that may be seen in congenital CMV infections were not observed in any patient.

Children in Group 1 were treated with ganciclovir (10 mg/kg/day, in two doses) for 21 days. Antipruritic treatment for cholestasis, calcium, vitamin D, and other lipophilic vitamin supplementation were given to both groups.

All the patients' clinical and laboratory parameters (age, icterus, organomegaly, ALT, AST, ALP, GGT, T. Bil., D. Bil., CMV Ig G and CMV Ig M antibody, CMV avidity and CMV-DNA) were evaluated initially and at the third month of the study. Anti-CMV IgG and IgM ab titers were measured with ELISA method and CMV-DNA was detected with PCR. CMV-DNA was purified with QIAmp DNA Blood Mini Kit (QIAGEN, Hamburg). Quantitative PCR analyses was performed by RealArt™ CMV RG PCR Kit used with Rotor-Gene™ 2000/3000. The quantitation standards were defined as copies/μl. An equation was used to convert the values determined using the standard curve into copies/ml of sample material:

Result (copies/ml):Result (copies/μl) × Elution volume(μl)Sample volume (ml)
 MathType@MTEF@5@5@+=feaafiart1ev1aaatCvAUfKttLearuWrP9MDH5MBPbIqV92AaeXatLxBI9gBaebbnrfifHhDYfgasaacH8akY=wiFfYdH8Gipec8Eeeu0xXdbba9frFj0=OqFfea0dXdd9vqai=hGuQ8kuc9pgc9s8qqaq=dirpe0xb9q8qiLsFr0=vr0=vr0dc8meaabaqaciaacaGaaeqabaqabeGadaaakeaacqqGsbGucqqGLbqzcqqGZbWCcqqG1bqDcqqGSbaBcqqG0baDcqqGGaaicqqGOaakcqqGJbWycqqGVbWBcqqGWbaCcqqGPbqAcqqGLbqzcqqGZbWCcqqGVaWlcqqGTbqBcqqGSbaBcqqGPaqkcqqG6aGodaWcaaqaaiabbkfasjabbwgaLjabbohaZjabbwha1jabbYgaSjabbsha0jabbccaGiabbIcaOiabbogaJjabb+gaVjabbchaWjabbMgaPjabbwgaLjabbohaZjabb+caViabeY7aTjabbYgaSjabbMcaPiabbccaGiabgEna0kabbccaGiabbweafjabbYgaSjabbwha1jabbsha0jabbMgaPjabb+gaVjabb6gaUjabbccaGiabbAha2jabb+gaVjabbYgaSjabbwha1jabb2gaTjabbwgaLjabbIcaOiabeY7aTjabbYgaSjabbMcaPaqaaiabbofatjabbggaHjabb2gaTjabbchaWjabbYgaSjabbwgaLjabbccaGiabbAha2jabb+gaVjabbYgaSjabbwha1jabb2gaTjabbwgaLjabbccaGiabbIcaOiabb2gaTjabbYgaSjabbMcaPaaaaaa@8C36@

For preparation of PCR assay, a standard volume (20 μl), of sample DNA was mixed with 30 μl of CMV RG Master. Data analyses was performed with Rotor-Gene™ software according to the manufacturer's manual.

Statistical analysis was performed using Mann-Whitney U Test for comparison of the initial and the third month changes of weight and CMV-DNA PCR, Fisher's Chi square exact test for the variants such as sex, organomegaly, jaundice and CMV antibody titers, and Wilcoxon Test was used for the comparison of individuals among each group (SPSS 13.0 version).

## Results

The mean age of seven babies (four female, three male) in Group 1 and five babies (one female, four male) in Group 2 was 3.7 ± 2.4 months and 6.6. ± 2.2 months, respectively. One baby in Group 1 and two babies in Group 2 had low weight percentile at the initial evaluation. There were no significant statistical difference between groups regarding to age, sex and weight (NS, NS, NS respectively).

In the physical examination, five babies in Group 1 and two in Group 2 had hepatomegaly (liver was palpated >2 cm below the last rib) at the first evaluation (NS). One baby in each group had palpable spleen evaluated as a normal variation.

Liver biopsy was performed for 7/7 babies in Group 1 and for 4/5 babies in Group 2. Although hepatic inclusion bodies are rarely found in the histopathologic examination of pediatric cases, it was detected in one baby in Group 1. Granulomatous changes and findings related to cholestasis were determined in four babies in the treatment group and two babies in the comparison group. Nonspecific histopathological findings of CMV hepatitis as lymphomonocytic cell infiltration, hydrophic degeneration, mild steatosis, perisinusoidal fibrosis, and Kuppfer cell hyperplasia were found in all babies.

At the initial evaluation, three babies in Group 1 and one baby in Group 2 had icterus. They were still icteric at the third month (NS) (Table 2, 3). In comparison with Group 2, initial levels of total bilirubin, AST, ALT, GGT, and ALP revealed a significant decrease in Group 1 after the treatment(p < 0.05) The differences of initial and third month values were statistically significant among the babies in Group 1 (Table 3). No significant change was observed in the comparison group at the third month regarding to initial values (NS) (Table 3). The serological evaluation for CMV revealed that all the babies were CMV IgG (+) and CMV IgM antibody was found in five and three babies in the treatment and comparison groups, respectively. CMV avidity test was not available for the whole group. The avidity value indicating infection longer than 3 months (avidity index >0.8) was determined in 60% and 33% of the evaluated babies in the treatment and the comparison group, respectively (Table 4). The confirmative serological changes were defined as the decrease in CMV IgG antibody or avidity titers, or loss of CMV-IgM antibody at the third month's evaluation. While all the patients in the treatment group had evidence of serologic improvement (p < 0.01), the comparison group did not reveal any significant change (NS). Similarly, CMV-DNA PCR values decreased to desirable levels following the treatment in Group 1 (p = 0.05). However, the changes in the comparison group were inconsiderable (Table 4).

## Discussion

Neonatal hepatitis refers to a group of pathologies causing similar morphologic changes in the liver of the babies less than three months of age. It is blamed for 40% of cholestatic situations in the neonates after exclusion of extrahepatic biliary atresia. It affects males more frequently than females, and similar results were found in the present study. As the term "idiopathic neonatal hepatitis" refers to the neonatal hepatitis of unknown (but probably multifactorial) etiology, "neonatal hepatitis" is caused by a group of well defined etiologic factors, so treatment should be considered [1,3,5].

Neonatal cytomegalovirus infection may occur due to either intrauterine or perinatal exposure to CMV infected cervicovaginal secretion and breast milk. The clinical spectrum of perinatal infection varies from an asymptomatic infection or a mild disease to severe systemic involvement, including central nervous system [6].

The clinical presentation of acute neonatal CMV infection resembles the mononucleosis of the Epstein-Barr virus seen in neonates and immunodeficient individuals with fever, malaise, and cervical lymphadenopathy. Severe jaundice and granulomatous hepatitis also have been established due to neonatal CMV infection [1,3]. Physical examination may reveal minimal hepatomegaly and mild jaundice, in addition to slightly increased serum aminotranspherases (less than threefold of normal values)[4].

CMV infection is unlikely to be a cause of massive hepatocellular necrosis in a normal host. Previous studies reported that transaminases reached the highest levels (<200 U) in the second or third week of infection, decreasing to normal values by the fifth week [4]. In our study, transaminases increased moderately in both groups, but a significant decrease at the third month was observed only in the treatment group.

The laboratory tests used for serologic diagnosis of CMV hepatitis are CMV-IgM ab, CMV early antigen (in tissue or blood), CMV-DNA PCR and virus cultures [8,12]. We could not obtain viral cultures or early antigen titers of the babies in our study, but the liver biopsy evaluations suggested CMV hepatitis. In the histopathologic examination of liver, the presence of cytomegalic cells and inclusion bodies refers to the intensive immune activation against viral attack. The liver damage in an immunocompetant individual is mostly due to the primary immune response of the host, whereas cytopathic damage of the virus has priority in patients suffering from immune deficiency [1].

Chang et al. recently evaluated the existence of CMV-DNA in liver biopsy samples of healthy neonates in comparison to the neonates with neonatal hepatitis. He reported that CMV DNA was detected in 46% of babies with neonatal hepatitis (n: 50) whereas none of the healthy group had viral DNA (n: 30). Thus, he suggested that CMV could play a major role in the pathogenesis of neonatal hepatitis [9]. Although it has been was reported that hepatomegaly might regress spontaneously in the first year of life in babies with congenital CMV infection, portal hypertension may occur without the evidence of cirrhosis [10,11]. In the current study, one patient in the comparison group died of abundant upper gastrointestinal haemorrhage at the age of 18 months as a result of portal hypertension without cirrhosis. Two patients in the same group also progressed to chronic hepatitis.

The necessity of treatment is controversial in neonatal CMV infection, as spontaneous recovery is expected in most cases unless severe systemic disease occurs [3,12]. However, as in the present study, an increasing number of studies indicate the necessity of treatment, especially in cases with symptoms of acute or chronic cholestatic hepatitis or proven histopathological findings [3,13-17]. A new study from Lanari et al. demonstrated the importance of high CMV-DNA titer on development of sequelaes. Furthermore, they suggested that the CMV-DNA quantity could be useful for identifying the patients who will benefit highly from antiviral therapy [18].

Ganciclovir is recommended as a first step antiviral agent for the management of congenital CMV infection. Most common adverse effects of ganciclovir treatment include dose-dependent neutropenia and blood counts including absolute neutrophil count. Therefore, leukocyte counts should be monitored closely during treatment. In our treatment group, we have not seen any severe adverse effects requiring the cessation of the treatment [3,6].

## Conclusion

We suggest that ganciclovir therapy significantly improves the clinical course of neonatal cholestatic CMV hepatitis. Currently the number of studies of neonatal cholestatic CMV hepatitis is insufficient. New and extensive research will lead us to clarify the efficacy of ganciclovir treatment in the early period of CMV hepatitis, and the preventive role of anti-viral therapy on progressive liver disease due to cholestasis and hepatitis in neonatal CMV infection.

## Abbreviations

CMV, cytomegalovirus; ALT, alanine aminotransferase; AST, aspartate amninotransferase; ALP, alkaline phosphatase; GGT, Gamma-glutamyl transpeptidase; T. Bil., total bilirubin; D. Bil., direct bilirubin; PCR, polymerase chain reaction.

## Competing interests

The author(s) declare that they have no competing interests.

## Authors' contributions

TBO and BD participated in the design of the study, patient selection and performed the statistical analyses.

RM participated in the design of the study and serologic evaluations.

HON performed the histopathologic examinations of the liver pathology specimen and collected data.

All authors read and approved the final manuscript.

## Pre-publication history

The pre-publication history for this paper can be accessed here:



## Figures and Tables

**Table 1:** The Demographic Characteristics of the Groups

**Groups** **(n = number of cases)**	**Age (months)** **Mean ± SD** ** Median**	**Sex**		**Weight (kg)** **Mean ± SD** ** Median**
			
		**M**	**F**	
Group 1 (n = 7)	*3.7 ± 2.4 3	** 3	4	△ 5.05 ± 1.6 4.7
Group 2 (n = 5)	*6.6 ± 2.2 7	** 4	1	△ 5.97 ± 2 5.9

*, **, △ NS

**Table 2:** The Comparison of the Physical Examination Findings of the Groups

**Groups**		**Jaundice** **(n)**	**Hepatomegaly** **(n)**
Group 1 (n = 7)	Initial	*3	**5
	Posttreatment	2	2
Group2 (n = 5)	Initial	*1	**2
	Control	2	2

*, ** NS

**Table 3:** The Comparison of the Biochemical Values of the Groups

**Groups**		**T. billirubin ** mean ± sd median	**D. Billirubin ** mean ± sd median	**ALT ** mean ± sd median	**AST ** mean ± sd median	**ALP ** mean ± sd median	**GGT ** mean ± sd median
Group 1 n = 7	Initial	*4.6 ± 3.2 4.6	*3.9 ± 2.5 4	*119.4 ± 56.4 80	*167 ± 68.4 197	*1148 ± 300 750	◆ 4414 ± 186.3 110
	Posttreatment	1.29 ± 0.82 2.5	0.53 ± 0.27 0.3	53 ± 18.5 54	75 ± 28.1 58	556 ± 196.5 324	150.7 ± 61.5 71
Group 2 n = 5	Initial	**2.52 ± 1.07 1.45	**1.42 ± 0.45 0.25	**172.7 ± 57.6 92	**155.2 ± 68 93	**393.5 ± 44.7 381	◆◆ 67.7 ± 25 49
	Control	4.37 ± 2.21 0.3	2.97 ± 1.75 0.1	123.2 ± 18.5 98	152.7 ± 47 116.5	435.7 ± 37.5 311	73.2 ± 24.6 51

Comparison of initial and post treatment values among Group 1

◆ p < 0.01

* p < 0.05

Comparison of initial and control values among Group 2

**, ◆◆ NS

**Table 4:** The Comparison of serological CMV markers in Groups

**Groups**		**◊CMV IgM (+) patient** **no/total**	**CMV IgG avidity >0,8 patient** **n**	* ΨCMV DNA **copy/ml**
Group 1 (n = 7)	Initial	7/7	5	2230(166–9240)
	Posttreatment	0/7	3	14 (8–1930)
Group 2 (n = 5)	Initial	3/5	3	2178(158–2917)
	Control	3/5	1	1915(656–2900)

◊ The ratio of patients, in each group, having affirmative results in the second evaluation (p < 0.01)

* Median (minimum-maximum)

Ψ The comparison of value changes between first and second evaluation (p < 0.05)
